# Research on gastrointestinal polyp detection method based on improved YOLOv7

**DOI:** 10.3389/fonc.2026.1827856

**Published:** 2026-06-10

**Authors:** Yiyan Zhang, Baojie Zhang, Ketao Ma, Yujie Chen

**Affiliations:** 1 School of Intelligent Manufacturing, Qingdao Huanghai University, Qingdao, China; 2 School of Physics and Electronic Information, Yantai University, Yantai, China

**Keywords:** deep learning, ECANet, EIoU, gastrointestinal lesion detection, YOLOv7

## Abstract

**Introduction:**

In the medical field, the detection of gastrointestinal polyps through endoscopic images is confronted with challenges such as complex backgrounds and numerous irrelevant factors. These issues often lead to a decrease in detection rates, an increase in missed detections, and a rise in the misdiagnosis rate of gastrointestinal lesions during early diagnosis.

**Methods:**

To enhance diagnostic accuracy, this paper proposes a gastrointestinal polyp detection method based on an improved YOLOv7 model. This method introduces the ECANet attention mechanism in both the head and neck of the YOLOv7 network structure to reduce the interference of image backgrounds and irrelevant factors, thereby improving the detection performance of the model. Furthermore, by replacing the loss function CIoU with EIoU, the improved model is able to better predict the bounding boxes of gastrointestinal polyps, getting closer to the real boxes, thereby enhancing the accuracy of model detection.

**Results:**

The models were compared on the Kvasir-SEG gastrointestinal polyp dataset. The precision of the improved model YOLOv7 (EIoU + ECANet) was 94%, the recall rate was 88.7%, and the mean average precision was 92.9%. Compared with the original model, all three indices have been improved.

**Discussion:**

The proposed YOLOv7 (EIoU + ECANet) model has strong robustness and generalization ability in the detection of gastrointestinal polyps.

## 1 Introduction

With the improvement of people’s living standards, dietary habits have undergone significant changes. Irregular and unhealthy eating habits have led to a continuous increase in the incidence of gastrointestinal diseases (1). The detection of gastrointestinal polyps has important clinical significance (2). Polyps are small protrusions formed in the gastrointestinal tract. They may be benign, but there is also a potential risk of malignancy. Gastrointestinal polyp detection is helpful in early identification of potential health issues, enabling effective intervention measures to be taken to safeguard the health and quality of life of patients.

The current methods for diagnosing gastrointestinal lesions mainly include X-ray barium meal, electronic endoscopic screening, tumor markers, routine blood tests, urea and breath tests (UBT). Due to its ability to clearly observe the entire gastric mucosa and intestinal mucosa of patients, electronic endoscopy makes it easier to determine the location, characteristics, and extent of lesions. Compared to other methods, it causes less damage to the body, and therefore becomes the preferred examination method for most patients (3, 4). To interpret endoscopic images to determine whether there are lesions in the gastrointestinal tract requires doctors to have profound professional knowledge and clinical experience. To deliver accurate diagnoses and timely treatment, doctors need to possess a deep understanding of different lesion features and skillfully assess their nature and extent. In endoscopic examinations, the experience of doctors plays a critical role in identifying small or early lesions (5). With the continuous advancement of artificial intelligence and big data technology, the utilization of machine vision technology based on deep learning (6) to extract features such as shape, color, and texture from images can effectively address the low accuracy and efficiency issues in the detection of gastrointestinal polyps using endoscopy. This approach serves as a potent method for tackling present challenges and represents a significant avenue of research aimed at advancing the sophistication of medical diagnosis and treatment. Presently, both domestic and international researchers have undertaken numerous studies focusing on the implementation of deep learning and machine vision technology in the realm of gastrointestinal lesions. Cao (7) and their research team applied the Single Shot MultiBox Detector (SSD) target detector in the detection of gastric polyps. They effectively improved the target detection performance by adjusting the backbone network, selecting EfficientNet23, tuning the loss function, and implementing improvements such as using Focal Loss and parametric rectified linear units as activation functions. Li (8) and others proposed an early gastric cancer detection model (EGCD) using a method based on Convolutional Neural Network (CNN) and attention mechanisms. They introduced an enhanced version of YOLOv4 by improving upon the YOLOv4 target detector and incorporating Convolutional Block Attention Module (CBAM) to enhance target feature expression. Long (9) et al. introduced the fully convolutional network (FCN) with the ability to automatically distinguish colon tissue from other backgrounds. This assists doctors in more effectively detecting and diagnosing intestinal diseases. Wen et al. (10) utilized transfer learning in conjunction with pre-trained models, conducted training on a large-scale image dataset and refined specific features of colonoscopy images. Their utilization of an enhanced FCN resulted in outstanding performance in segmenting polyps within colonoscopy images.

Currently, there are two common issues in the detection of gastrointestinal polyps, namely, insufficient attention to key feature information by the models and inadequate consideration of the relationship between target ground-truth boxes and predicted boxes, which may lead to inaccurate predictions. To address these issues, this paper proposes a method for detecting gastrointestinal polyps based on an improved model of YOLOv7 (11). YOLOv7 has a relatively strong ability to detect small targets, real-time processing performance, and adaptability to medical image diagnosis, making it particularly suitable for addressing key issues such as the variable sizes and real-time requirements in polyp detection. Therefore, this paper takes YOLOv7 as the basic model for improvement. Expanding upon the framework of YOLOv7, this model incorporates the ECANet attention mechanism into both the neck and head regions. This mechanism allows for the adaptive adjustment of channel feature weights, enabling the network to better focus on the characteristics of polyp lesions, suppress interference from complex backgrounds and irrelevant information, and enhance the detection performance of the model. Furthermore, the model utilizes the Efficient Intersection over Union (EIoU) loss function to evaluate the distance between predicted boxes and ground-truth boxes. This improves the accuracy of box detection, enhancing the overall performance of the model. Finally, the improved model is validated on the Kvasir-SEG dataset, demonstrating its effectiveness in gastrointestinal polyp detection. This approach aims to address the challenges of insufficient feature attention and inaccurate box predictions in gastrointestinal polyp detection, providing a more accurate and efficient method for identifying these lesions.

## 2 Methods

### 2.1 Data preprocessing

**Figure 1:**
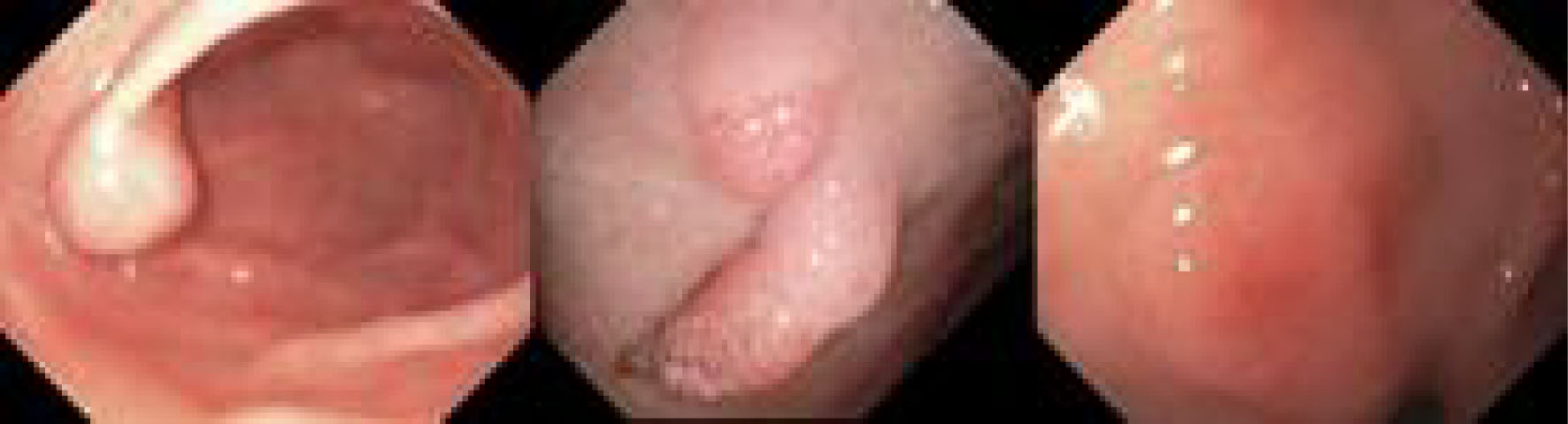
Images of gastrointestinal polyps.

The object of target detection in this study is gastrointestinal polyps. Research has found that early detection of colorectal cancer has a significant impact on patient survival rates, making timely detection of polyps particularly crucial. In colonoscopy examinations, polyps are often prone to being overlooked, leading to miss rates ranging from 14% to 30%, depending on the type and size of the polyp. Increasing the detection rate of polyps has been shown to effectively reduce the risk of developing colorectal cancer (12). Therefore, enhancing the timely detection of polyps not only helps improve treatment outcomes but also has a positive impact on the overall health status of patients. A common image of a polyp is shown in **Figure 1** below.

Ensuring the quality of the dataset is crucial for the robustness and generalization of the model after training. The quality of the dataset directly impacts the model’s resilience and applicability when faced with new data. Therefore, a high-quality dataset can provide the model with more accurate and comprehensive information, helping to train a more robust and generalizable model. The dataset for this study was selected from the Kvasir-SEG dataset of gastrointestinal polyps. The Kvasir-SEG dataset adopts implicit data partitioning. The official has not clearly stated the basis for the split (880:120). In practice, it has been found that some images have been split into the training/validation sets, which may lead to data leakage. In view of this, we used the 880 images as the original data in the study. This study randomly selected 100 original images as the test set, 200 original images as the validation set, and the remaining 580 original images were augmented to 700 as the training set. Through data augmentation operations such as rotation, translation, brightness adjustment, lowering brightness, as well as introducing Gaussian noise and salt and pepper noise (13) to the collected image data, the dataset can be effectively expanded and enriched. The 1000 research images with annotated gastrointestinal polyp lesions were manually labeled using the labeling tool, and their positional information was saved as txt files. Subsequently, the dataset was divided into training, validation, and testing sets with a ratio of 7:2:1 for model training and testing purposes. **Table 1** provides detailed information on the gastrointestinal lesion polyp image dataset:

**Table 1:** Dataset information.

Dataset	Number of images
Training set	580→700
Validation set	200
Test set	100
Total images	1000

### 2.2 Improvements to YOLOv7

#### 2.2.1 YOLOv7 model

The YOLOv7 model has significant advantages in the field of object detection (14). Compared to its predecessor YOLOv5 (15), this model not only maintains its speed advantage but also improves detection accuracy by enhancing the backbone network and using feature fusion methods to achieve higher detection accuracy. By employing techniques such as SPP-PANet for multi-scale feature fusion and adaptive convolution, YOLOv7 achieves faster detection speeds while maintaining high detection accuracy. Its architecture is relatively simple, making it easy to expand and modify, and it provides practical tools and interfaces for users to quickly train and deploy models. Additionally, YOLOv7 performs excellently on large-scale, multi-class datasets and demonstrates effective training and detection capabilities on small-sample, small-class datasets. YOLOv7 has been improved and optimized based on the previous YOLO series algorithms, with the network structure divided into three parts: backbone, neck and head (16, 17).

##### 2.2.1.1 Backbone

The core function is to extract multi-scale features layer by layer from the input image, converting the original pixels into feature maps rich in semantics. The backbone consists of three parts: Convolutional Backbone Structure (CBS), Efficient Layer Aggregation Network (ELAN), and Max Pooling Module (MP). Specifically, the Convolutional Backbone Structure (CBS) includes 2D Convolution (Conv2d), Batch Normalization (BN), and Silu activation function. The main role of the ELAN module is to extract image features and control the number of network channels. Meanwhile, MP-1 consists of a Maxpool layer and CBS, used to reduce the size of feature maps, thus reducing the network’s parameter and computational demands (18).

##### 2.2.1.2 Neck

The core function is to integrate the multi-scale features output by the Backbone, enhance the interaction of information across different levels, and improve the detection ability for targets of different sizes. This section consists of the MP-2, Spatial Pyramid Pooling Cross Stage Partial Connections (SPPCSPC), and ELAN-H modules. The SPPCSPC module design enables the network to obtain various receptive fields, effectively discriminating between large and small objects. This design also mitigates the high computational expenses associated with consecutive stacks of convolutional layers (19).

##### 2.2.1.3 Head

The core function is to directly predict the bounding box, category and confidence of the target based on the feature map obtained after Neck fusion.

#### 2.2.2 ECANet attention mechanism

Due to the complex background of gastrointestinal endoscopy images and the influence of gastrointestinal light on endoscopic images, detecting gastrointestinal polyps becomes more challenging. Traditional methods reduce the interference of complex backgrounds on polyp lesion detection in original images, but their efficiency is relatively low. The model should reduce attention to irrelevant feature information without the need to balance among all information in the image. Therefore, this model introduces an attention mechanism based on YOLOv7. The attention mechanism is a widely used data processing method in machine learning, especially in the field of computer vision. It enhances the weight of specific information by giving the model attention to different parts, thus optimizing task execution. Efficient Channel Attention Network (ECANet) (20) introduces a channel attention mechanism to capture channel relationships and enhance feature representation capabilities. It dynamically tunes the weights of channel features, emphasizing crucial aspects while dampening insignificant ones. This process effectively fortifies the network’s representation and prevents unnecessary parameters and computational expenses.

**Figure 2:**
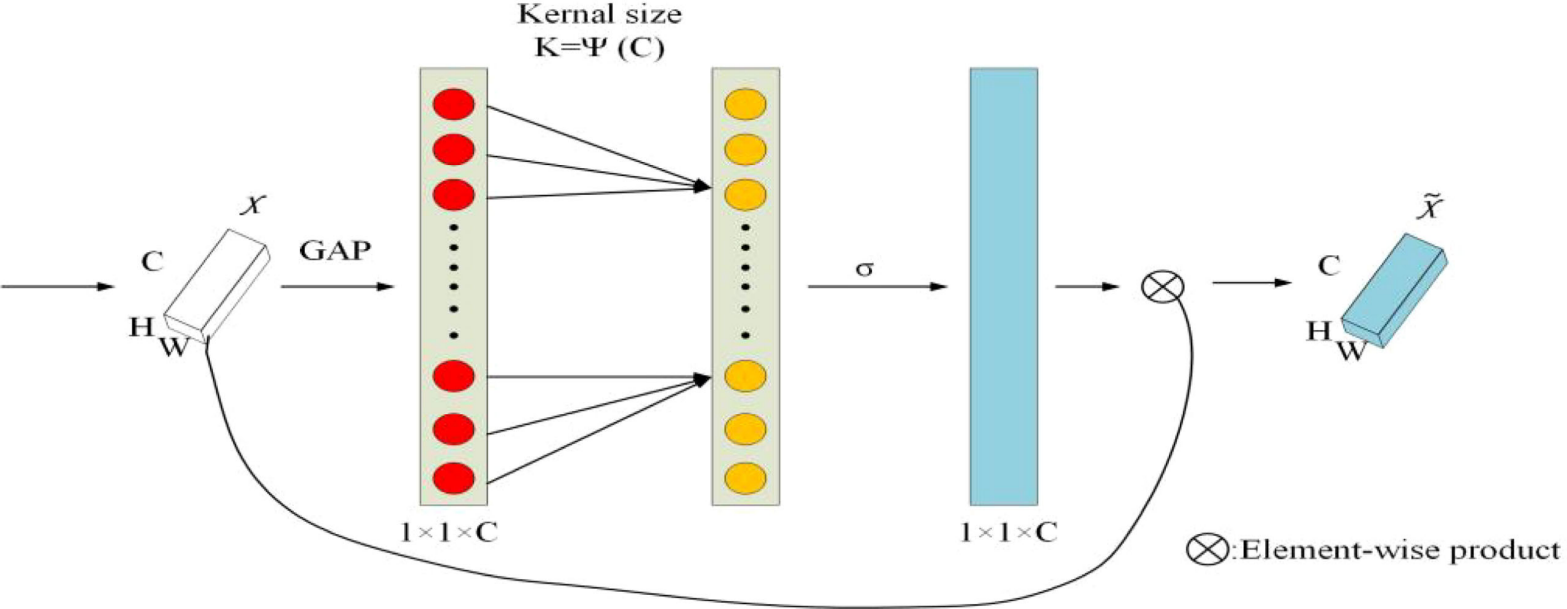
ECANet attention mechanism.

ECANet improves upon the two main issues of the traditional SENet, proposing “de-fully-connected + adaptive one-dimensional convolution”, achieving superior performance without significantly increasing parameters or computational costs. The basic structure is shown in **Figure 2**.

The Channel Attention Module is a core component of ECANet, designed to enhance feature representation by adaptively adjusting channel feature weights. The module takes feature maps as input, computes the global average pooling to obtain the global average value for each channel, and then generates channel attention weights through a fast one-dimensional convolution. These weights are applied to each channel to achieve a weighted combination of different channels. Finally, the adjusted features are normalized by a scaling factor to maintain their range.

The ECA Attention Module effectively captures the interaction of information, where the range of interaction is determined by the size *k* of the one-dimensional convolutional kernel. Manually adjusting the kernel size *k* would require a significant amount of computational resources. Therefore, the kernel size *k* can be automatically adjusted based on its mapping relationship with the channel size *C*, as shown in Equation 1.

(1) C=ϕ(k)

Due to the limited representation of relationships by linear functions, and because the channel size *C* is often set as a power of 2, linear functions can be expanded into nonlinear functions, as shown in Equation 2. Therefore, it can be understood that when a channel size *C* is given, the kernel size *k* can be adaptively determined by Equation 3.

(2) $$C=\phi (k)=2^{\left(\gamma *k-b\right)}$$

(3) $$k=\psi (C)={\left|\frac{\log_{2}C}{\gamma }+\frac{b}{\gamma }\right|}_{odd}$$

In the expression, ${\left|*\right|}_{odd}$ represents the nearest odd number to *. Given γ=2 and b=1.

#### 2.2.3 Improvement of loss function

In model training, the role of the loss function is crucial. It quantifies the performance of the model by evaluating the distance between predicted boxes and ground-truth boxes, where a smaller distance indicates a smaller loss function value. The choice of a suitable loss function for a specific detection problem has profound effects on the convergence speed, localization accuracy, and final performance of the model. In object detection algorithms, intersection over union (IoU) (21) is an essential component of many loss functions, used to measure the similarity between predicted boxes and ground-truth boxes. IoU represents the ratio of the area of overlap between the target ground-truth box and the model predicted box to the total area covered by the two boxes. The IoU can be calculated based on Equations 4 and 5.

(4) $$IoU=\frac{\left|B\cap B_{i}\right|}{\left|B\cup B_{i}\right|}$$

(5) $$L_{IoU}=1-IoU=1-\frac{\left|B\cap B_{i}\right|}{\left|B\cup B_{i}\right|}$$

In the formulas, *B* represents the area of the target ground-truth box, *B_i_* represents the area of the model predicted box, and *L_IOU_* is bounded between 0 and 1.

When the ground-truth box and the predicted box do not overlap completely, the IoU loss cannot effectively reflect the relationship between them, making model training difficult. The complete intersection over union (CIoU) loss function addresses this by introducing a correction term, providing a more comprehensive measure of the distance between the predicted box and the ground-truth box. This allows the model to better grasp the object’s positional and shape details across various overlapping scenarios, thereby enhancing the efficiency of training.

The CIoU loss function is a type of loss function used in object detection. It considers the center point distance, aspect ratio, and overlap area information between two bounding boxes, aiming to make object bounding box regression more stable and accurate. Specifically, the formula for the CIoU loss function is Equation 6, the parameters α and v can be calculated using Equation 7 and 8.

(6) $$L_{CIoU}=1-IoU+\frac{\rho^{2}(b,b^{gt})}{c^{2}}+\alpha v$$

(7) $$\alpha =\frac{v}{(1-IoU)+v}$$

(8) $$v=\frac{4}{\pi^{2}}{\left(\arctan\frac{\omega^{gt}}{h^{gt}}-\arctan\frac{\omega }{h}\right)}^{2}$$

These formulas include several variables: *h* represents the height of the predicted box; *ω* represents the width of the predicted box; represents the height of the ground-truth box; $\omega^{gt}$ represents the width of the ground-truth box; *b* represents the center point of the predicted box; *b^gt^* represents the center point of the target box; $\rho^{2}$ represents the Euclidean distance between the center points of the two boxes; *c* represents the diagonal distance of the minimum closed bounding rectangle enclosing both boxes.

Zhang et al. proposed the Efficient Intersection over Union (EIoU) loss function (22). EIoU further improves upon CIoU by decoupling the aspect ratio constraint into two independent penalty terms for width and height, thereby enhancing the convergence speed and regression accuracy. It consists of three parts, including IOU loss, center point distance loss, and width-height loss. The objective of the width-height loss is to directly minimize the differences in width and height between the target box and the anchor box. The calculation method for EIoU loss can be obtained from Equations 9 and 10.

(9) $$EIoU=IoU-\frac{\rho^{2}(b,b^{gt})}{c_{\omega }^{2}+c_{h}^{2}}-\frac{\rho^{2}(\omega ,\omega^{gt})}{c_{\omega }^{2}}-\frac{\rho^{2}(h,h^{gt})}{c_{h}^{2}}$$

(10) $$L_{EIoU}=1-EIoU$$

In the calculation of the bounding box regression loss, the EIoU loss function is chosen for improving the model by addressing some issues with other loss functions and demonstrating good performance. Here, *c_h_* represents the height of the minimum enclosing box covering the predicted box and the ground truth box, while $c_{\omega }$ represents the width of the minimum enclosing box covering the predicted box and the ground truth box.

**Figure 3:**
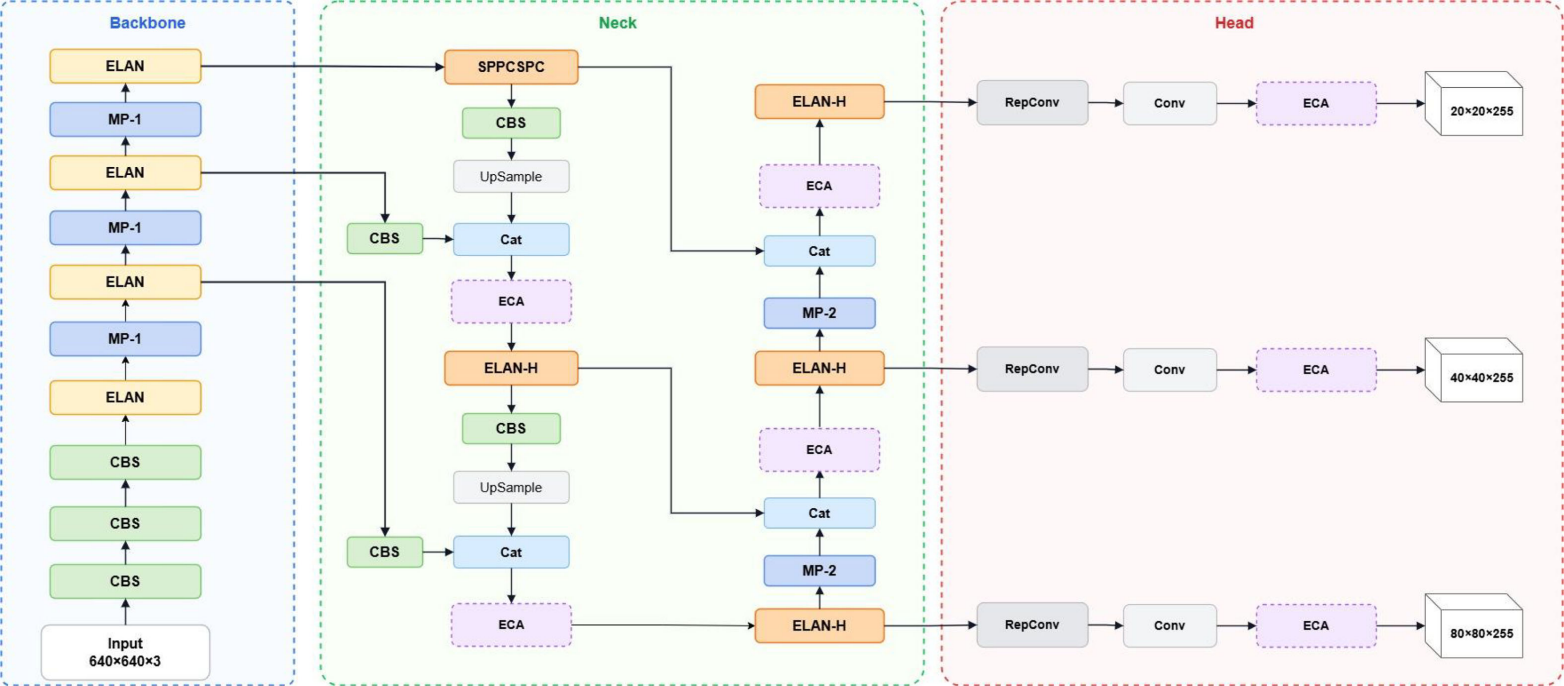
The structure diagram of the improved YOLO v7.

The structure diagram of the improved YOLO v7 proposed in this paper is shown in **Figure 3**.

## 3 Results and discussion

### 3.1 Experimental environment and evaluation metrics

The experiment was conducted on a 64-bit Windows 11 system, with the hardware platform consisting of an AMD Ryzen 7 7735H with Radeon Graphics @ 3.20 GHz CPU and an NVIDIA GeForce RTX 4060 GPU with 8GB of VRAM. The software environment used was Python 3.8 and Pytorch 2.1.1, with GPU acceleration enabled using CUDA 11.8 and CUDNN 8.7.0. The experimental data came from the Kvasir-SEG dataset, comprising a total of 1,000 images, with 700 images in the training set, 200 in the validation set, and 100 in the testing set.

In the detection of gastrointestinal polyp lesions, this study employed precision (*P*), recall (*R*), and mean average precision (*mAP*) as performance evaluation metrics.

### 3.2 Experimental comparison and analysis

To verify the superiority of the proposed improved YOLOv7 model in this paper, comparative experiments were conducted in the same experimental environment using the same dataset. Firstly, the YOLOv7 model proposed in this paper was compared with YOLOv7 models incorporating attention mechanisms CBAM, Channel Attention (CA), Squeeze-and-Excitation (SE) and ECA. Additionally, one layer of ECA attention mechanism was added to the neck, and ECANet attention mechanisms were introduced to the head and neck separately for comparison. Then, the best-performing models were compared by replacing the generalized intersection over union (GIoU), EIoU, and wise intersection over union (WIoU) loss functions.

#### 3.2.1 Testing results of YOLOv7 with different attention mechanisms

**Table 2** shows the comparison of YOLOv7 with different added attention modules. The addition of different attention modules sometimes resulted in improved performance compared to the original YOLOv7 model, while in other cases, the performance decreased. Notably, adding CA and ECA attention modules resulted in significant increases in *P*, *R*, and *mAP*. The most significant improvement was observed in networks where ECA attention modules were added to the neck and head separately. *P* increased by 9 percentage points, *R* increased by 5.5 percentage points, and increased by 9.7 percentage points compared to the original model. This indicates that adding attention mechanisms after improvement allows the model to better assess the weight distribution of different features, resulting in more accurate object detection results.

**Table 2:** Comparison of test results of different attention mechanisms.

Models	P	R	mAP
YOLOv7	83.1%	82.4%	82.0%
YOLOv7 with ECA in Neck	90.1%	86.0%	88.0%
YOLOv7 with ECA in Neck and Head	**92.1%**	**87.9%**	**91.7%**
YOLOv7 with CBAM	62.9%	69.0%	59.0%
YOLOv7 with CA	91.0%	86.4%	91.0%
YOLOv7 with SE	78.8%	82.4%	83.3%

Bold: indicates the best performance in the comparison.

#### 3.2.2 Testing results of YOLOv7 with different loss functions

The original YOLOv7 model used the CIoU loss function. From **Table 3**, It is evident that models incorporating GIoU and EIoU exhibit a notable increase in *P*. Among these, the EIoU improvement is the most prominent, showing an increase of 4.1 percentage points. This indicates that the EIoU loss function is more accurate in calculating the distance relationship between predicted boxes and real boxes.

**Table 3:** Comparison of test results of different loss functions.

Models	P	R	mAP
YOLOv7	83.1%	82.4%	82.0%
YOLOv7(EIoU)	**87.2%**	**83.3%**	**84.1%**
YOLOv7(GIoU)	84.1%	82.7%	83.2%
YOLOv7(WIoU)	78.2%	65.3%	65.3%

Bold: indicates the best performance in the comparison.

#### 3.2.3 Testing results of YOLOv7 with simultaneous improvement of loss functions and attention mechanisms

**Figure 4:**
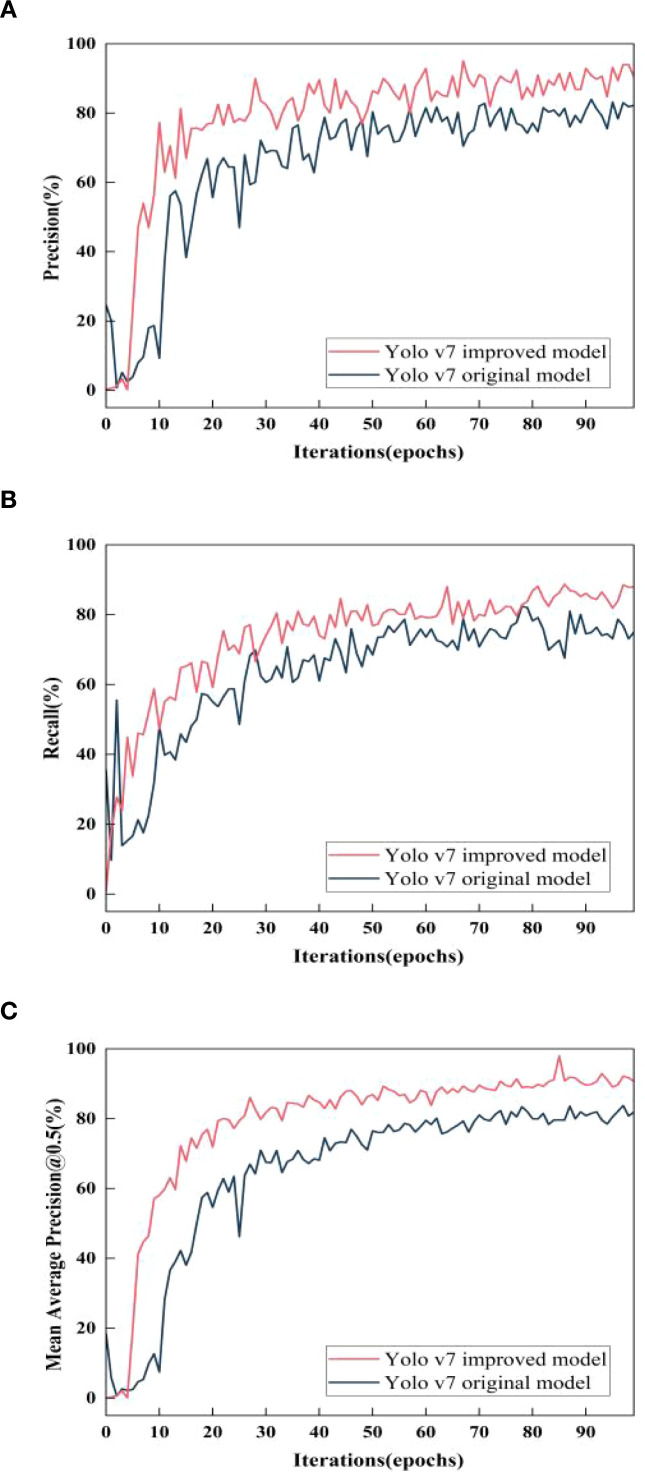
Comparison of model performances. **(a)** Comparison of Precision. **(b)** Comparison of Recall. **(c)** Comparison of Mean Average Precision.

Comparative experiments were conducted on the improvement of loss functions and attention mechanisms in **Tables 2**, **3**. The results showed that adding ECA attention mechanisms to the neck and head and using EIoU as the loss function in the original network yielded the best results. After 100 iterations of training, the improved model achieved *P*of 94%, *R* of 88.7%, and *mAP* of 92.9%. As can be seen in **Table 4**, the improved YOLOv7 network showed the most significant advantage compared to the original network, with *P* increasing by 8.9 percentage points, *R* increasing by 6.3 percentage points, and *mAP* increasing by 10.9 percentage points. This was the best-performing model among all experimental groups. The performance comparison between the improved and original models in various aspects is shown in **Figure 4**.

**Table 4:** Comparison of test results with simultaneous improvement of loss functions and attention mechanisms.

Models	P	R	mAP
YOLOv7	83.1%	82.4%	82.0%
YOLOv7 with ECA in Neck and Head (EIOU)	**94.0%**	**88.7%**	**92.9%**
YOLOv7 with ECA in Neck and Head (WIOU)	55.1%	83.9%	53.7%
YOLOv7 with ECA in Neck and Head (GIOU)	88.9%	84.3%	88.5%

Bold: indicates the best performance in the comparison.

In conclusion, this article focuses on two main improvements to YOLOv7:

- The ECANet attention mechanism was introduced into the head and neck parts of the YOLOv7 network to reduce the interference of image backgrounds and irrelevant factors, thereby improving the detection performance of the model.
- Replacing the CIOU loss function with EIOU, the improved model can more accurately predict the bounding boxes of gastrointestinal polyps, get closer to the ground truth boxes, and thereby enhance the detection accuracy of the model.

In this study, we made targeted improvements to YOLOv7 and achieved effective optimization of precision and recall in the Kvasir-SEG dataset, which is consistent with the current research trend in the field of intelligent diagnosis of gastrointestinal diseases. Chou et al. used the spectrum-aided vision enhancer (SAVE) mechanism to convert traditional white light endoscopy (WLI) images into simulated hyperspectral imaging (HSI) and narrow band imaging (NBI) images to improve the classification accuracy of gastrointestinal diseases (23, 24). Unlike the idea of them, this study focuses on directly optimizing the internal structure and feature extraction ability of the target detection model, thereby enhancing the sensitivity of lesion recognition. Moreover, compared with Sahoo et al. extensively evaluated the applicability of the YOLOv11 series models in colonoscopy polyp detection (25), although this study is based on the earlier YOLOv7 architecture, it has achieved competitive detection performance on the Kvasir-SEG dataset through deep model improvement strategies, demonstrating the important research value and application potential of refining existing mature architectures. Although this study is limited to a single public dataset, future verification of the generalization ability of the model on different types, multi-center data, and real-time video streams is still needed. However, the proposed improvement scheme in this study provides a high-precision, low-false-negative rate computer-aided diagnostic tool for clinical use, which is of significant clinical application value for verifying endoscopic reports, improving the reading efficiency of endoscopists, and achieving precise identification of early digestive tract lesions.

## 4 Conclusion

This study aims to improve the YOLOv7 model used for detecting gastrointestinal lesions, with a focus on optimizing the loss function and integrating the attention mechanism. The evaluation results show that the improvements made to the YOLOv7 model significantly enhance the effect of feature extraction and bounding box regression, which improves the accuracy and robustness of lesion detection. The study found that using image enhancement techniques on the public dataset helps reduce the risk of overfitting in the training data, thereby improving the reusability and efficiency of the data. This research was conducted only on a single public dataset Kvasir-SEG. In the future, diverse datasets such as NBI and video data will be integrated to include various types of gastrointestinal lesions. The evaluation indicators used in this study are mainly precision and recall, in order to reduce missed diagnosis and false diagnosis. In the subsequent studies, evaluations in terms of parameter quantity and real-time performance will also be included. Additionally, future research will explore lightweight network architectures to deploy the model on embedded platforms.

## Data Availability

The original contributions presented in the study are included in the article/supplementary material. Further inquiries can be directed to the corresponding author.
